# Supplementary material for: Fatty Acid Profiles of Some Siberian Bryophytes and Prospects of Their Use in Chemotaxonomy
Source: Biomolecules. 2023 May 15;13(5):840. doi: 10.3390/biom13050840 (PMC10216562; doi:10.3390/biom13050840)
Supplement: Supplementary file 1 [file biomolecules-13-00840-s001.zip › biomolecules-2220816-supplementary.pdf]

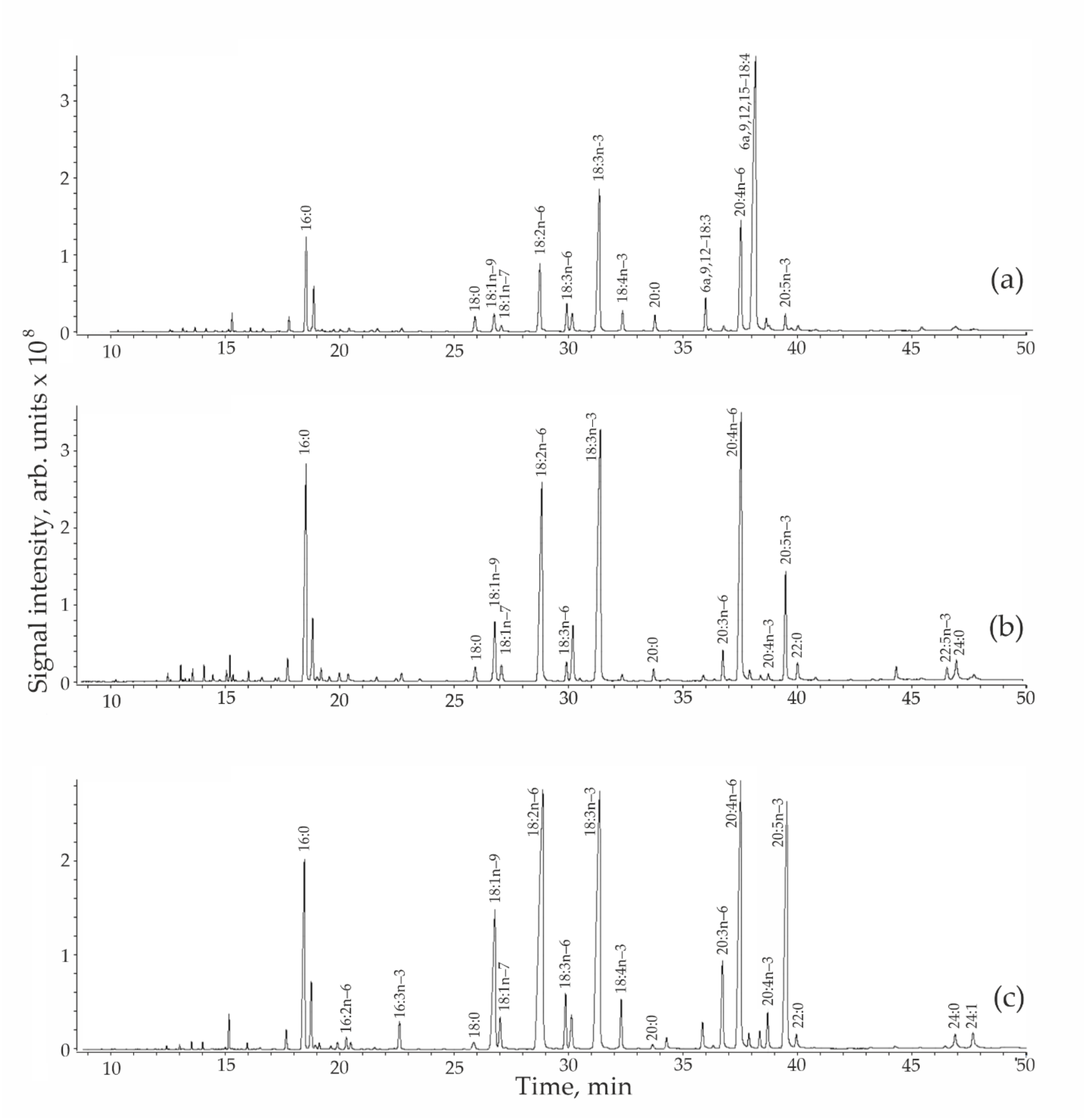

Figure S1. Examples of chromatograms of methyl esters of fatty acids including two acetylenic acids, octadeca-9,12-dien-6-ynoic (6a,9,12–18:3) and octadeca-9,12,15-dien-6-ynoic (6a,9,12,15–18:4), and some rare fatty acids (20:4n–3, 22:5n–3) found in bryophytes (a) *Dicranum* spp., (b) *Thuidium assimile*, (c) *Rhytidium rugosum*.

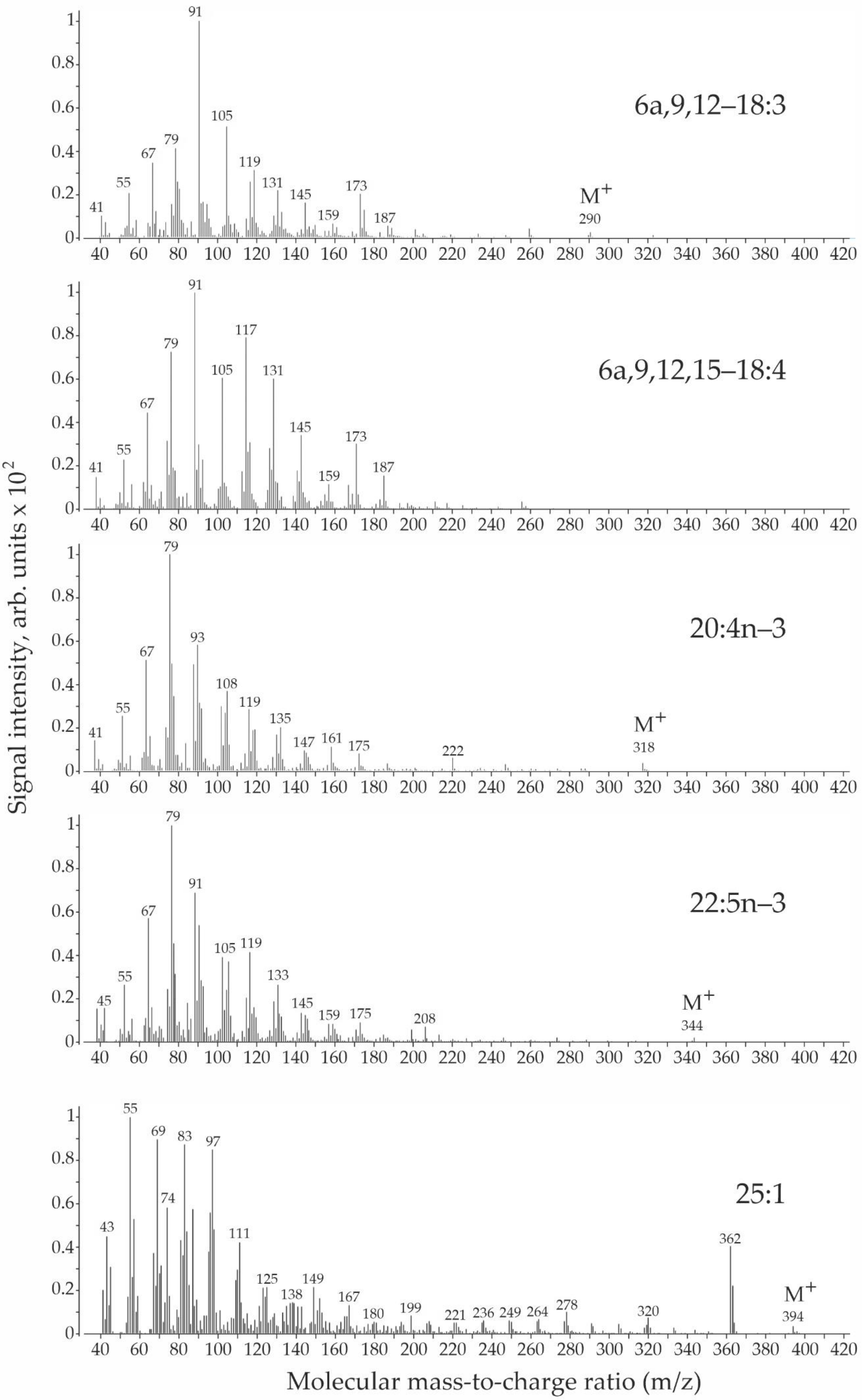

6a,9,12–18:3
6a,9,12,15–18:4
20:4n–3
22:5n–3
25:1
M+
290
318
344
394
Signal intensity, arb. units x 10^2
Molecular mass-to-charge ratio (m/z)

Figure S2. Mass spectra of two acetylenic acids, octadeca-9,12-dien-6-ynoic (6a,9,12–18:3) and octadeca-9,12,15-dien-6-ynoic (6a,9,12,15–18:4), two polyunsaturated fatty acids, 20:4n–3 and 22:5n–3, and one monounsaturated fatty acid, 25:1. M⁺—molecular ion.

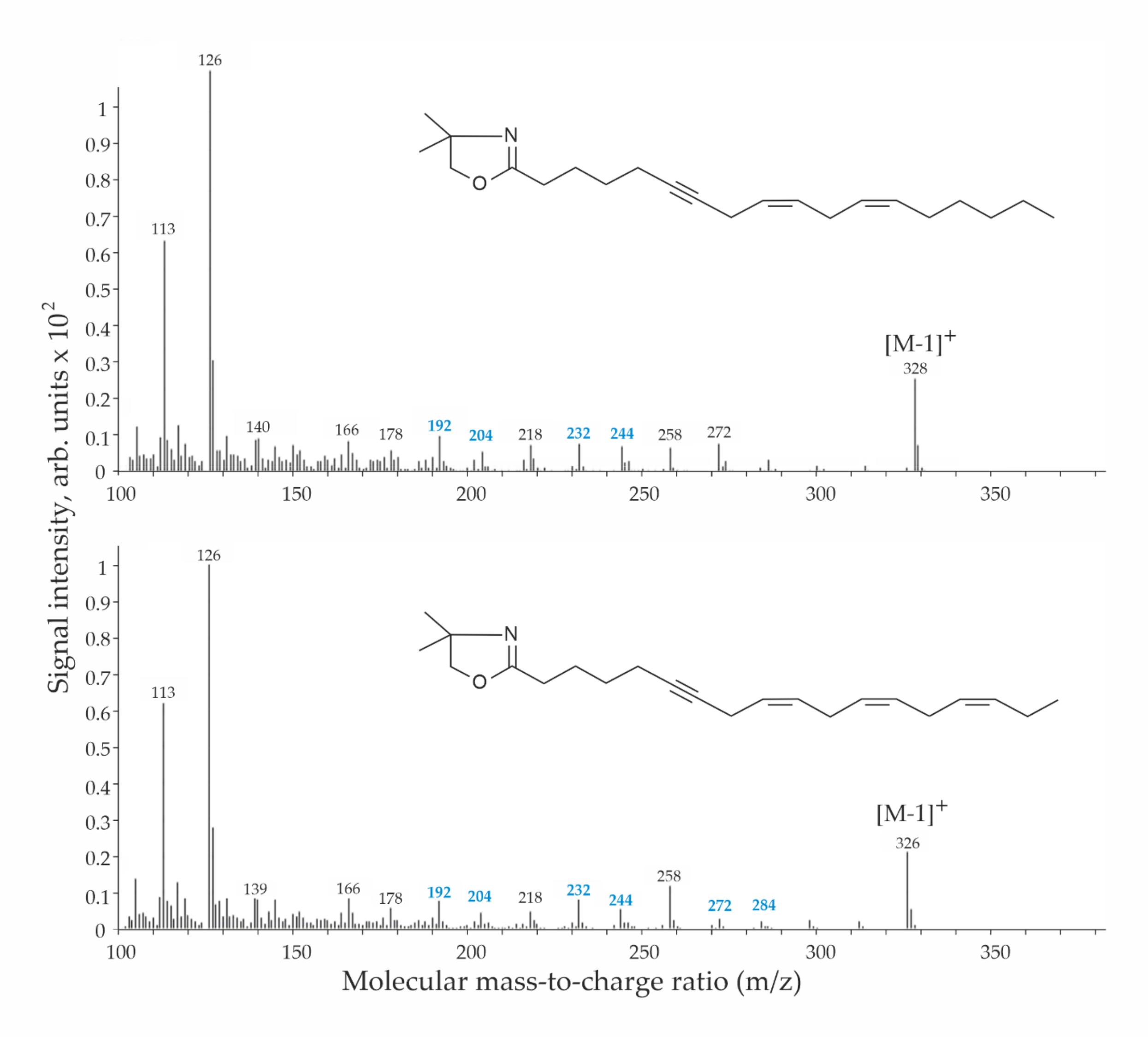

Figure S3. Mass spectra of dimethyloxazolic derivatives of two acetylenic FA: octadeca-9,12-dien-6-ynoic (6a,9,12–18:3) and octadeca-9,12,15-dien-6-ynoic (6a,9,12,15–18:4). Blue numbers show diagnostic fragments. [M-1]⁺—molecular ion minus one atomic mass.
